# Visceral Injuries in Patients with Blunt and Penetrating Abdominal Trauma Presenting to a Tertiary Care Facility in Karachi, Pakistan

**DOI:** 10.7759/cureus.3604

**Published:** 2018-11-17

**Authors:** Bushra Kiran Naeem, Sughra Perveen, Nadia Naeem, Tanweer Ahmed, Iqbal Khan, Imran Khan, Muhammad Tahir, Mazhar Iqbal

**Affiliations:** 1 General Surgery, Jinnah Post Graduate Medical College, Karachi, PAK; 2 Miscellaneous, Dow University of Health Sciences (DUHS), Karachi, PAK; 3 General Surgery, Jinnah Postgraduate Medical Centre, Karachi, PAK; 4 General Surgery, Jinnah Postgraduate Medical Center, Karachi, PAK; 5 General Surgery, Jinnah Postgraduate Medical College, Karachi, PAK

**Keywords:** abdominal trauma, blunt, penetrating, visceral injuries, tertiary care, pakistan, abdominal injury, visceral injury, blunt and penetrating abdominal trauma

## Abstract

Introduction

Abdominal injuries are responsible for 10% of the mortalities due to trauma. Delays in early diagnosis or misdiagnoses are two major reasons for the mortality and morbidity associated with abdominal trauma. The objectives of this study were to determine the frequency of visceral injuries in patients with abdominal trauma and compare the frequency of visceral injuries in patients with blunt and penetrating abdominal trauma.

Methods

We conducted a cross-sectional study from May 2016 to May 2018 of patients presenting to the emergency department (ED) at Jinnah Postgraduate Medical Center in Karachi, Pakistan. Patients were 12 to 65 years old and presented within 24 hours of abdominal trauma. We recorded the type of abdominal visceral injuries, such as liver, spleen, intestine, stomach, mesentery, and pancreas.

Results

The mean patient age was 31 ±13 years. Penetrating trauma was found in most patients (n=72, 51%). Liver injuries were found in 37 patients (26.4%), spleen injuries in 29 patient (20.7%), stomach injuries in eight patients (5.7%), intestine injuries in 67 patients (47.9%), mesentery injuries in 21 patients (15%), and pancreas injuries in nine patients (6.4%). The type of abdominal trauma was found significantly associated with liver injury (p-value 0.021), and intestine injury (p-value <0.001).

Conclusion

Penetrating trauma (51.4%) was more common than blunt trauma (48.5%), and intestines are the most commonly affected by penetrating and blunt trauma injuries (70.1% and 47.8%, respectively). The liver is the most commonly affected (42.85%) in blunt trauma injuries, followed by the spleen (28.5%). The appropriate authorities should consider this information when instituting public health and safety initiatives.

## Introduction

Trauma is damage to the body caused by an exchange with environmental energy that is beyond the body’s resilience [1]. Civilian trauma is one of the major causes of death worldwide. Technological advances in diagnostic measures have led many trauma surgeons to implement conservative management [1]. Based on the mechanism of trauma, abdominal trauma is classified into two categories, penetrating trauma and blunt trauma [2]. Penetrating abdominal trauma may involve peritoneal breaches, such as stabbing and gunshot wounds. Blunt abdominal trauma (BAT) refers to road traffic injuries and injuries due to falls where impact or countercoup wounds enter the peritoneal cavity and are more common than penetrating abdominal trauma [2]. The most affected organs are solid, and the treatment approach is conservative in these cases. Hollow visceral lesions are difficult to diagnose [3]; incorrect diagnoses in patients with hollow visceral injuries occur in up to 45% of cases [4].

The goals of our study were to determine the frequency of visceral injury in patients with abdominal trauma and compare the frequency of visceral injuries in patients with blunt and penetrating abdominal trauma. Many trauma victims are referred to Jinnah Hospital’s emergency department daily secondary to car, bicycle, firearm, stab, and fall injuries. Ours is the largest tertiary care hospital in Karachi, Pakistan, and, currently, no local data exist on the frequency of abdominal visceral injuries in patients with blunt and penetrating trauma. We hope the outcomes of our study will provide practical, usable data to help policymakers and lawmakers make informed policy decisions in the interest of public safety.

## Materials and methods

This cross-sectional, observational study was conducted in the surgery department at Jinnah Postgraduate Medical Centre, Karachi, Pakistan, from May 2016 to May 2018.

Patients over the age of 12 years, presenting during the study duration, which commenced from May 2016 and ended in May 2018, with blunt or penetrating abdominal trauma, were included to be the part of the study. A total of 140 participants were included.

Polytrauma patients with neurological, limb, and/or thoracic trauma were excluded from the study.

We defined BAT as presentation with abdominal pain over a score of five on the visual analog scale, tenderness on physical examination, and presenting to the emergency department due to any road injury or fall. All three parameters were required to qualify as BAT. Penetrating abdominal trauma was defined as presenting with an open wound in the abdominal area and presenting to the emergency department due to any firearm injury or stab assault. Abdominal visceral injuries were defined as injuries to the spleen, liver, stomach, intestine, mesentery, pancreas, or kidney.

After receiving approval from the hospital’s ethics committee to conduct the study and oral and written informed consent from patients and/or guardians, data were collected from all qualifying cases. Participants were informed of the purposes, procedures, confidentiality measures, risks, and benefits of the study prior to providing consent. We also collected basic demographic data consisting of age, gender, trauma type, and duration of trauma.

After receiving initial treatment, patients were sent to the radiology department for an immediate evaluation of their abdominal visceral injuries by a chest X-ray (erect posteroanterior view), abdominal X-ray (erect and supine), focused abdominal sonography for trauma (FAST), and computed tomography (CT) scan.

We conducted an exploratory laparotomy in all penetrating trauma patients and BAT patients with clinical signs of peritonitis, tenderness, those who were hemodynamically unstable, or if the FAST revealed free fluid. Data regarding abdominal visceral injuries (i.e., injuries to the liver, spleen, intestine, stomach, mesentery, and pancreas) as per the operational definition were noted and recorded on a special proforma. Injuries were graded according to the American grading system [5].

We used IBM SPSS Statistics for Windows, Version 22.0 (IBM Corp., Armonk, NY, US) for statistical data analysis. The frequency and percentage data were computed for qualitative variables, such as gender, type of abdominal trauma, and abdominal visceral injuries to the liver, spleen, intestine, stomach, mesentery, and pancreas. We determined the mean ± standard deviation for quantitative variables such as age and duration of trauma.

Patients with blunt and penetrating abdominal trauma were compared in terms of visceral injuries by applying the chi-square test; p ≤ 0.05 was considered statistically significant.

Stratification was done for effect modifiers (i.e., age, gender, and duration of trauma) to see their effect on abdominal visceral injuries. A post-stratification chi-square test was applied; p ≤ 0.05 was considered statistically significant.

## Results

The mean age of the study participants was 31 ± 13 years (Table 1).

**Table 1:** Percentage of visceral injuries

Visceral injuries	Blunt trauma 68 (48.5%)	Penetrating trauma 72 (51.4%)	Total (%)
	n (%)	n (%)	
Intestine	20 (35.7%)	47 (70.1%)	67 (47.8%)
Liver	24 (42.85%)	13 (19.40%)	37 (26.4%)
Spleen	16 (28.5%)	12 (17.91%)	28 (20.0%)
Kidney	12 (21.4%)	11 (16.4%)	23 (16.4%)
Pancreas	4 (7.1%)	5 (7.4%)	9 (6.4%)
Stomach	2 (3.5%)	6 (8.96%)	8 (5.71%)

**Figure 1:**
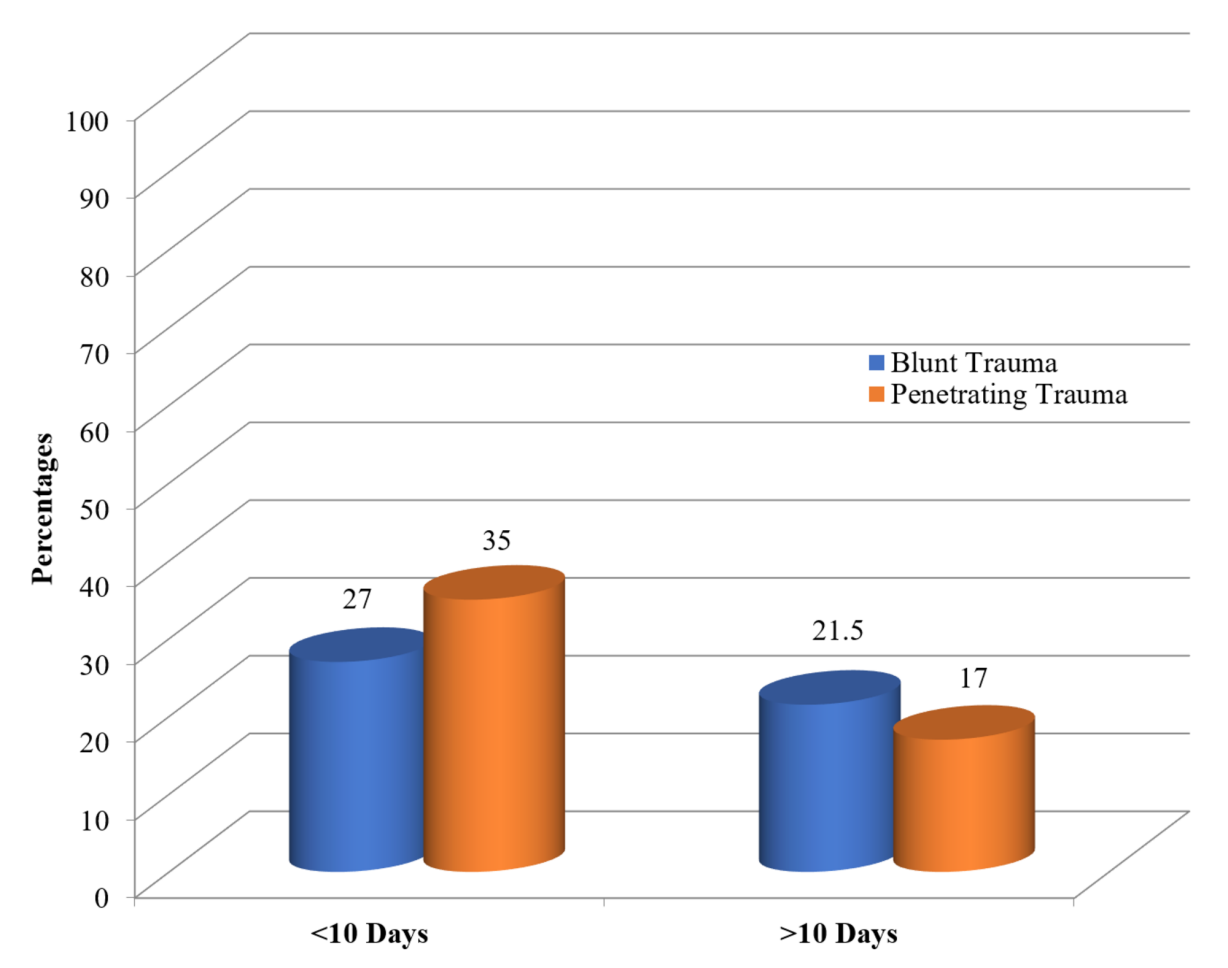
Length of stay

Of the 140 participants involved, 75 patients (53.5%) were younger than 30 years. Most of the participants were male (88.6%; n = 124), only 16 were female (11.4%). The age range with the most frequent trauma presentation is from age 15 to 70 years, which indicates that abdominal trauma is more prevalent in younger patients. The male-to-female ratio in our study was 9:1. Penetrating trauma is more common in male patients (88.5%, n = 124/140) as compared to female patients (11.5%, n = 16). The hospital length of stay in our study was 10 days, and BAT was associated with longer stays due to the involvement of multiple visceral injuries (Figure 1).

The liver was the most commonly injured organ in cases of BAT (42.85%), followed by the intestines and spleen (35.7% and 28.5%, respectively). Nearly half of our study participants had BAT (48.5%; n = 68), and penetrating trauma occurred in 51.5% (n = 72). Penetrating trauma is only slightly more common than BAT. Road traffic injury (RTI) was the most common cause of BAT (58%), followed by falls from a height (32%) and assaults (10%).

The intestines were most commonly impacted by penetrating trauma (70.1% of cases), followed by the liver and spleen (19.4% and 17.9%, respectively). The most common mode of injury in penetrating trauma cases was gunshot (85%) and stab wounds (15%). In penetrating hollow viscous injuries, the liver was the most frequently injured organ followed by the spleen.

A grade 4 liver trauma was most common in our study, likely due to the high incidence of RTIs and the forceful decelerating injuries of fixed organs in blunt trauma. Penetrating injuries had a relatively equal distribution of all grades. Splenic injuries were commonly Grades 2 and 3 in penetrating injuries; blunt trauma injuries were usually Grades 3 and 4. Pancreatic injuries were rare, and most were Grade 3 or 4.

Liver injuries were significantly associated with blunt trauma (p = 0.021) and intestinal injury were significantly associated with penetrating trauma (p <0.001). Age and gender shared no significant association with visceral injuries (p > 0.05).

## Discussion

Many less affluent countries like Pakistan have a significant proportion of road and industrial trauma in a generally young population. The mortality and morbidity associated with trauma, either blunt or penetrating, can be reduced by early and effective medical intervention [1]. In Pakistan, gunshot injuries and other penetrating trauma are largely due to various terrorist attacks, and hospitals, especially tertiary care centers, accept the burden of these incidents.

In large cities like Karachi, a growing population, greater numbers of motor vehicles in use, and heavy traffic increase the risk of road traffic injuries (RTI). The incidence of robbery and social aggression also correlate to a higher incidence of penetrating trauma [6].

Several of our findings align with findings in previous reports, including the male-to-female ratio [7], penetrating trauma is more common in males [8], RTI as the most common cause of BAT [1-2,9], length of hospital stay [7], and BAT is associated with longer hospital stays [10].

Many of our findings did not align with previous reports. Our mean age was 10 years lower than the mean age reported by Costa et al. [7]. While intestines were most often injured in our study, Abri et al. reported liver, spleen, and kidneys were injured more often than intestines [8]. In cases of BAT, we found the liver was the most commonly injured organ, but other reports found the spleen to be the most commonly injured organ [11-12]. Our study’s incidence of blunt trauma was higher than in previous reports [1-2,13], and we found that gunshot wounds were the most common cause of penetrating injury while previous reports indicate stab wounds were the more common cause of penetrating injury [4,8,13]. The easy access to illegal weapons in our society has caused an increase in the number of gunshot injury cases and, therefore, governing bodies should take steps to address this concerning trend.

The literature lacks consensus on the hierarchy of organs injured in penetrating hollow viscous injuries [1,8,13-14].

Our study was limited in that we were unable to cover other tertiary care setups in Karachi. The correct percentages of blunt and abdominal trauma cannot be thus reported. Trauma victims who died immediately were not included in this study, as the patient could not present to the surgical team. Further research is required to analyze the actual number of cases presenting with blunt and penetrating abdominal trauma.

## Conclusions

We found penetrating trauma was more common than blunt trauma and the intestines are the most commonly affected by penetrating and blunt trauma injuries. For blunt trauma injuries, the liver is the most commonly affected, followed by the spleen. The liver, as the largest organ, is more liable to injury. Given this information, steps must be taken by the governing bodies to decrease these incidences in the future for the safety of the population.
